# LipidSnapshot – Treatment gaps in hypercholesterolemia in patients with atherosclerotic cardiovascular disease documented by office-based cardiologists and general practitioners in Germany

**DOI:** 10.1007/s00392-025-02751-z

**Published:** 2025-08-28

**Authors:** Oliver Weingärtner, Simon Glück, Karl Werdan, Jessica Schorr, Daniel Thieme, Ana de la Llave, Christian von Vultée, Winfried Haerer

**Affiliations:** 1 Department of Internal Medicine I, Division of Cardiology, Angiology and Intensive Medical Care, Friedrich Schiller University, University Hospital Jena, Am Klinikum 1, 07747 Jena, Germany; 2 Center for Health Services Research of the German Cardiac Society (DGK-ZfKVF), German Cardiac Society, Duesseldorf, Germany; 3 BNK Service GmbH, Munich, Germany; 4 Department of Internal Medicine III, University Hospital Halle (Saale), Martin Luther University Halle-Wittenberg, Halle (Saale), Germany; 5 Novartis Pharma GmbH, Nuremberg, Germany; 6 Real World Solutions, IQVIA, Frankfurt/M, Germany; 7 Cardiologicum Herzklinik Ulm MVZ, Ulm, Germany

**Keywords:** Hypercholesterolemia, Atherosclerotic cardiovascular disease, Lipid lowering therapy, Gender disparity, Age disparity, Non-interventional study

## Abstract

**Aims:**

Office-based cardiologists (OBCs) and general practitioners (GPs) follow different approaches for hypercholesterolemia management in atherosclerotic cardiovascular disease (ASCVD). This study evaluates whether differences in clinical practice between OBCs and GPs contribute to existing gaps in low-density lipoprotein cholesterol (LDL-C) control and lipoprotein(a) [Lp(a)] screening in ASCVD care.

**Methods:**

LipidSnapshot is a collaborative research initiative comprising a prospective non-interventional study at OBCs and a retrospective analysis of GP records. It evaluates LDL-C target attainment, Lp(a) testing, and lipid-lowering therapies (LLT) in the OBC and the GP setting. Subgroup analyses by gender and age are conducted.

**Results:**

The dataset comprises 1,500 ASCVD patients from OBCs and 82,375 patients from GPs. The median LDL-C levels were 68 mg/dL (OBC) vs. 88 mg/dL (GP). LDL-C targets < 55 mg/dL were achieved in 27.4% of patients (OBC) vs. 12.1% of patients (GP). Lp(a) testing rate was 20.3% (OBC) vs. 3.0% (GP). The proportion of patients not receiving any LLT was 1.5% (OBC) vs. 26.6% (GP). LDL-C levels were numerically higher in female patients as well as in younger patients especially in the GP setting. Female patients were less likely to receive LLT compared to their male counterparts and half of the GP patients < 50 years of age remained untreated at all.

**Conclusion:**

A large proportion of ASCVD patients in Germany are inadequately treated, with notable differences between GPs and OBCs. Additionally, gender and age-related disparities are evident. There is a clear need for these gaps to be addressed to improve cross-sectional patient care.

**Graphical Abstract:**

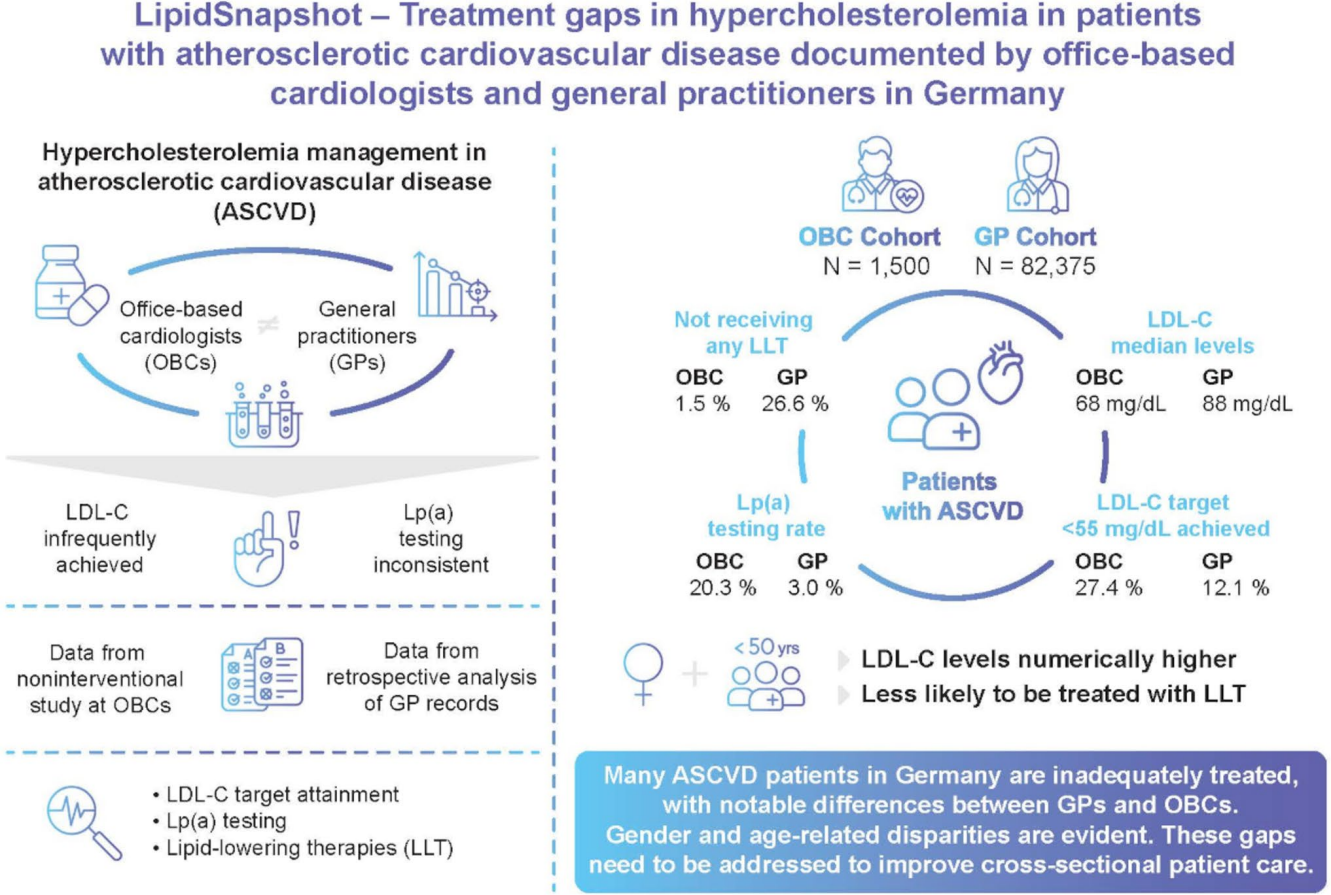

## Introduction

Cardiovascular disease (CVD) is the leading cause of death, accounting for approximately 40% of deaths in Europe in 2019 and 34% of deaths in Germany in 2022 [1]. In comparison with other European and non-European high-income countries (i.e., France, Switzerland, Spain, Japan), Germany and the United Kingdom, along with the USA, perform below average in terms of life expectancy, despite a cost-intensive, high-standard healthcare system. For Germany, in particular, the data suggests that the unfavourable pattern of cardiovascular mortality may be driven by underperforming primary care and disease prevention [2]. Elevated low-density lipoprotein cholesterol (LDL-C) levels are known to be a major risk factor for the development of atherosclerotic CVD (ASCVD) [3]. Elevated Lipoprotein(a) [Lp(a)] is an inherited, independent, and causal driver of CVD risk [4]. The reduction of elevated LDL-C levels is an important element in the primary and secondary prevention of cardiovascular events. Several treatment options are currently available to reduce LDL-C levels, whereas elevated Lp(a) levels cannot yet be treated pharmacologically.

In Germany, the primary specialties for the management of hypercholesterolemia are office-based cardiologists (OBCs) and general practitioners (GPs). However, different guideline recommendations for the management of these patients are available. The NVL (Nationale Versorgungsleitlinie) Version 6.0 represents one of the principal guidelines for GPs [5]. It references the “fire-and-forget” strategy based on the recommendations of the DEGAM (German Society of General Practitioners). In addition, the NVL refers to the “treat-to-target” strategy, as recommended by the DGIM (German Society of Internal Medicine) and the DGK (German Cardiac Society) among others. Lp(a) testing is not endorsed by the NVL. In the NVL Version 6.0 the LDL-C target level was 70 mg/dL [5]. The DGK, which provides guidance to German cardiologists, has adopted the 2019 European Society of Cardiology/European Atherosclerosis Society (ESC/EAS) guidelines for the management of dyslipidemia [6]. In this context, the ESC/EAS guidelines give a class I recommendation (evidence level A) for a treatment goal in patients with ASCVD defined as a reduction of LDL-C by a minimum of 50% from the baseline level with an LDL-C target level of < 55 mg/dL (< 1.4 mmol/L). Furthermore, the guidelines give a class IIa recommendation (evidence level C) that Lp(a) testing should be considered at least once in an individual’s lifetime to identify those with particularly elevated Lp(a) levels [6]. Even though general practitioners and internists, or cardiologists, may advocate divergent treatment strategies, there is a consensus that all ASCVD patients should be prescribed statin therapy [5, 6].

A simulation study indicated that an LDL-C target level of < 55 mg/dL could be achieved in more than 90% of patients with current lipid-lowering drugs [7]. The study “Jena auf Ziel – JaZ” also corroborated the feasibility of the LDL-C target within the German healthcare context. In this study, all high-risk patients achieved the recommended LDL-C targets following combination therapy with high-intensity statin and ezetimibe, or, if necessary, after therapy escalation with bempedoic acid or proprotein convertase subtilisin/kexin type 9 inhibitors (PCSK9i) [8]. Twelve months after the index event, patients enrolled in the JaZ study (“Jena auf Ziel”) had the option of either continuing with regular follow-up in the outpatient lipid clinic or transitioning to standard care by their GP. A further twelve-months follow-up revealed that patients in the outpatient lipid clinic had a significantly longer time on LDL-C target compared with patients treated by GPs (82.4% vs. 62.4%). The main factor contributing to the failure of LDL-C target attainment was the termination or alteration of lipid-lowering therapy (LLT) by GPs [9].

These findings are consistent with those of the DA VINCI study, a European cross-sectional observational study on the management of dyslipidaemia. The results demonstrated that the ESC/EAS targets are achieved in routine care in only a small proportion of patients (approximately a quarter in secondary prevention), primarily due to incomplete utilization of the available therapies [10]. Even in the cohort of young patients at risk, more than half of the patients did not achieve the LDL-C targets, the available therapies were not exhausted and a relevant proportion of the young patients did not even receive any LLT at all, as demonstrated in another cross-sectional study [11]. Moreover, the EUROASPIRE V cross-sectional study revealed a gender disparity in the management of risk factors for cardiovascular events, with women exhibiting a lower likelihood of attaining LDL-C targets [12].

LipidSnapshot aims to assess the implementation of the latest evidence-based ESC/EAS guidelines for the management of dyslipidaemia with updated LDL-C targets and Lp(a) testing guidance in OBC and GP settings in Germany. In addition, the project will analyse whether gender- and age-specific differences in hypercholesterolemia management exist in these settings.

## Methods

### Study design

The LipidSnapshot project represents a unique collaborative research effort between the Centre for Health Services Research of the German Cardiac Society (DGK-Zentrum für Kardiologische Versorgungsforschung, DGK-ZfKVF), the Federal Association of Cardiologists in Private Practice (Bundesverband niedergelassener Kardiologen, BNK), the German Society for Lipidology (Deutsche Gesellschaft für Lipidologie e. V., DGFL – Lipid-Liga), and Novartis Pharma.

The study is comprised of two parts, each of which will collect data from three annual snapshots between the years 2023 (snapshot 1), 2024 (snapshot 2) and 2025 (snapshot 3). Part 1 of the respective snapshot is a prospective, non-interventional, multicentre study at OBCs across Germany with 1,500 patients with ASCVD. Part 2 of the respective snapshot is a retrospective, aggregated analysis of anonymous electronic medical records of 250,000 ASCVD patients with a comparable dataset to OBC patients documented by GPs using the IQVIA Disease Analyzer. In both parts, adult male and female patients with a diagnosis of coronary artery disease (CAD), peripheral artery disease (PAD), a history of myocardial infarction (MI) or ischemic stroke with documented LDL-C levels may be enrolled. Relevant ICD-10 codes are summarized in Table 1. For both parts, repeated inclusion of individual patients is not mandatory.

**Table 1:** Relevant ICD-10 codes for inclusion of patients

Abbreviation	Definition	ICD-10 Code
SAP	Stable Angina pectoris	I20.1, I20.8, I20.9
USAP	Unstable Angina pectoris	I20.0
MI	Myocardial infarction	I21, I22, I23, I24.1, I25.2
PCI	Percutaneous coronary intervention (Angiography)	Z95.1, Z95.5
IS	Ischemic stroke	I63, I64, I69.3, I69.4
TIA	Transient cerebral ischaemic attacks	G45
PAD	Peripheral artery disease	I70.2
I25	Chronic ischaemic heart disease	I25 excluding I25.2

The data presented here are derived from the initial snapshot 1. OBC data was collected in August 2023, GP analysis included data documented between July 2022 and June 2023. Aggregated and anonymized data sets included diagnosis (with corresponding ICD codes), age, sex, LDL-C levels (mandatory), and Lp(a) levels (if available), and LLT by category.

### Objectives

The primary objective of the initial snapshot was to assess the proportion of ASCVD patients documented by OBCs compared to GPs achieving the 2019 ESC/EAS defined LDL-C treatment goal of < 55 mg/dL. Secondary objectives included the proportion of ASCVD patients with Lp(a) testing, LDL-C levels, Lp(a) levels, LDL-C levels by category, and the description of LLT. Subgroup analyses were performed to identify potential differences in the management of ASCVD patients based on gender and age.

### Statistics

Data has been analysed descriptively. Categorical variables were summarized using frequency counts and percentages. Continuous variables were summarized as median together with Q1 and Q3 as well as minimum and maximum. No formal statistical tests were performed for group comparisons.

## Results

### Demographics and disease characteristics

The data set of part 1 comprised 1,500 patients with ASCVD, documented by 49 OBCs. Part 2 comprised 237.810 patients with ASCVD documented by 996 GPs, with available LDL-C levels for 82,375 patients, who were thus included in the analysis. Patients treated by OBCs were on average 72.4 years old and 75.8% were male, while patients in the IQVIA dataset were on average 72.5 years old and 60.5% were male. The age distribution was balanced between OBC- and GP-documented patients (Table 2). The medical history with respect to concomitant CV-relevant diseases is presented in Table 2.

**Table 2:** Demographics and disease characteristics

	OBC N = 1,500	GP N = 82,375
**Gender, %**		
Female	24.2	39.5
Male	75.8	60.5
**Age in years**		
Mean (SD)	72.4 (10.0)	72.5 (11.9)
**Age in years by category, %**		
18–49	1.8	3.3
50–59	8.6	11.1
60–69	27.7	24.5
70–79	33.4	28.0
≥ 80	28.5	33.0
Missing	0.0	0.1
**CV-relevant disease history, %**		
Dyslipidaemia	96.2	51.0
PCI/CABG	60.7/21.1	9.7
Prior myocardial infarction	45.0	17.2
Diabetes mellitus	29.9	41.4
Peripheral arterial occlusive disease	11.1	8.9
Ischemic stroke	8.6	18.9

*CABG*, coronary-artery bypass grafting; *CV*, cardiovascular; *GP*, general practitioners; *OBC*, office-based cardiologists; *PCI*, percutaneous coronary intervention; *SD*, standard deviation

### Lipid profile

**Fig. 1:**
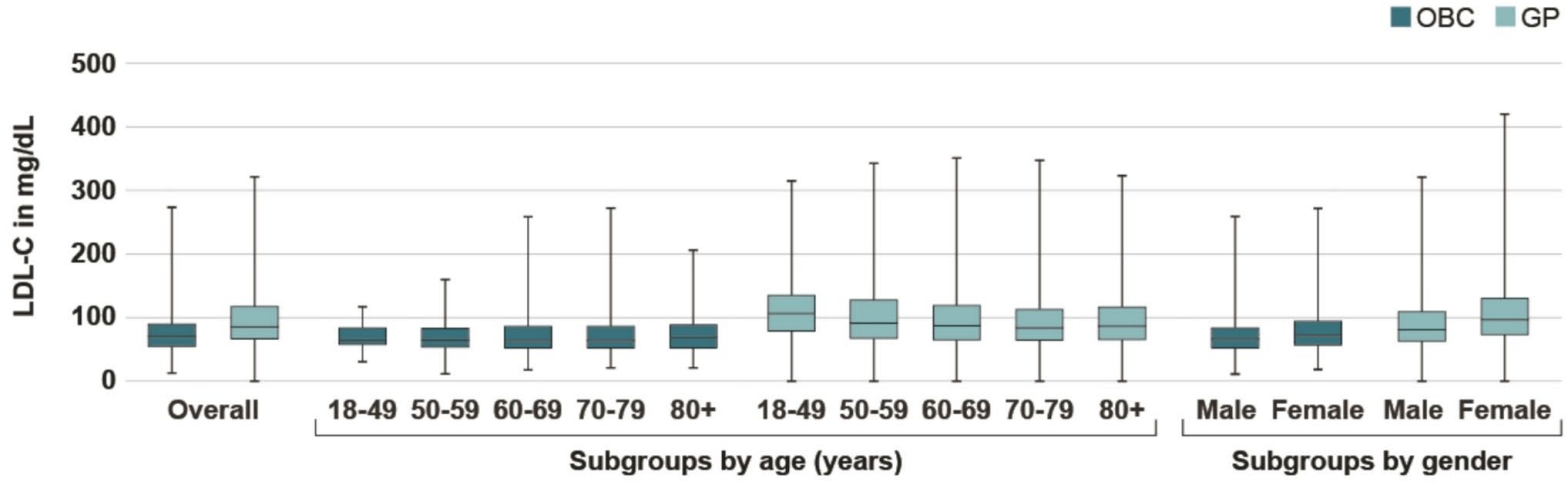
Box plots of median LDL-C levels in mg/dL (boxes indicate Q1 and Q3, whiskers indicate minimum and maximum values) in the overall OBC and GP cohorts as well as by age subgroups and by gender subgroups

The median LDL-C levels were 68 mg/dL in the OBC cohort compared to 88 mg/dL in the GP cohort. In the age subgroups, the median levels of LDL-C ranged from 65 to 71 mg/dL in the OBC cohort, with the highest value observed in the age group + 80 years, the median levels of LDL-C ranged from 85 to 106 mg/dL with the highest value observed in the age group 18–49 years (Fig. 1). Median LDL-C levels in men and women were 66 mg/dL vs. 73 mg/dL in the OBC cohort and 82 mg/dL vs. 98 mg/dL in the GP cohort (Fig. S).

**Fig. 2:**
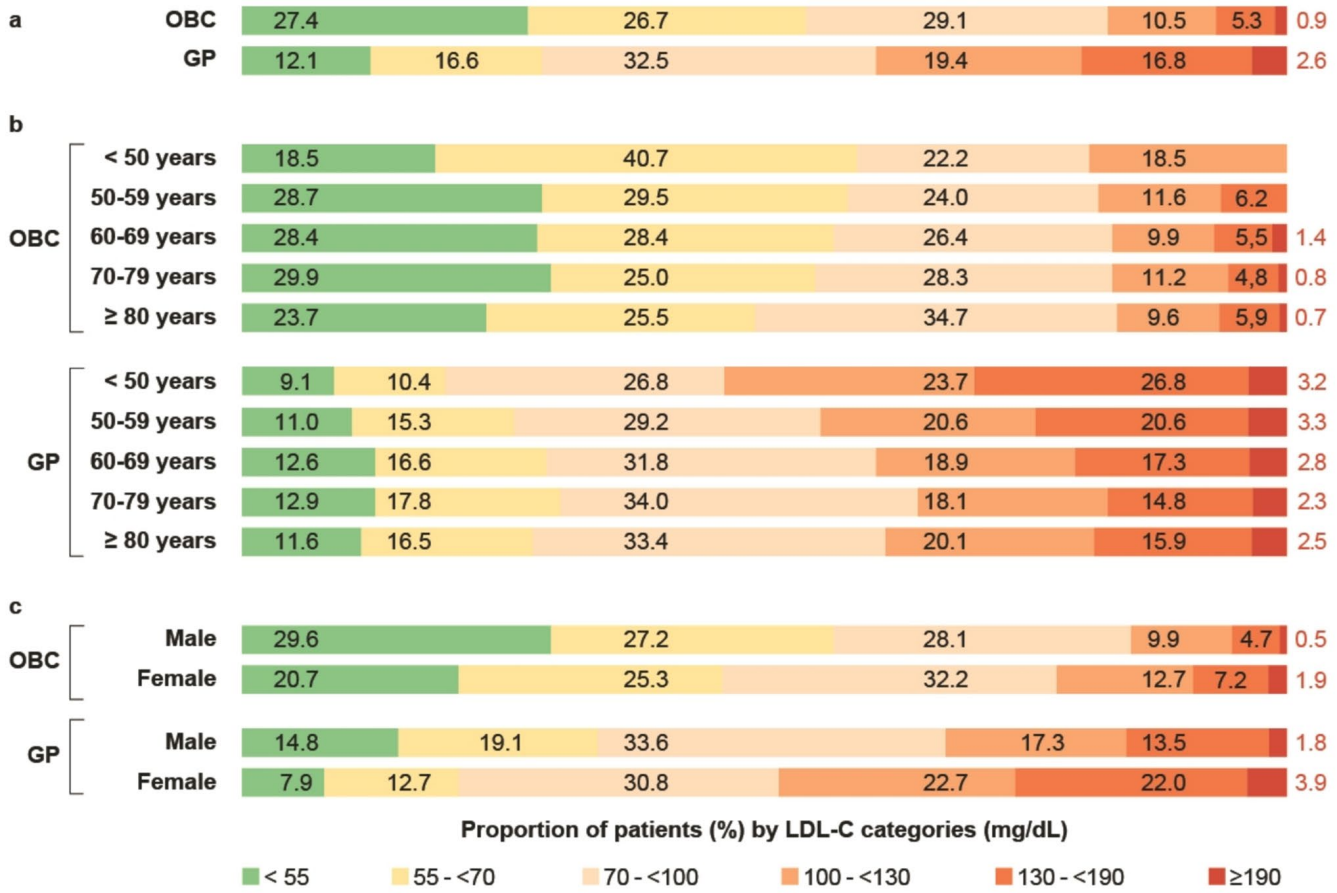
Proportions of patients by LDL-C category in the total OBC and GP cohorts [a], in the OBC and GP cohorts by age subgroup [b], and in the OBC and GP cohorts by gender subgroup [c]

The LDL-C target of < 55 mg/dL was achieved in 27.4% of patients in the OBC cohort and 12.1% of patients in the GP cohort. LDL-C targets of < 70 mg/dL were achieved in 54.1% and 28.7% of patients, respectively (Fig. 2a).

An analysis of target achievement by age showed that in the OBC cohort, 20 to 30% of patients in all categories achieved LDL-C targets of < 55 mg/dL, including patients younger than 50 years. In the GP setting, 10 to 13% of patients achieved the LDL-C target of < 55 mg/dL (Fig. 2b).

The LDL-C target achievement rates in male and female patients were 29.6% vs. 20.7% in the OBC cohort and 14.8% vs. 7.9% in the GP cohort for levels < 55 mg/dL and 56.8% vs. 46.0% in the OBC cohort and 33.9% vs. 20.6% in the GP cohort for LDL-C targets of < 70 mg/dL (Fig. 2c).

**Fig. 3:**
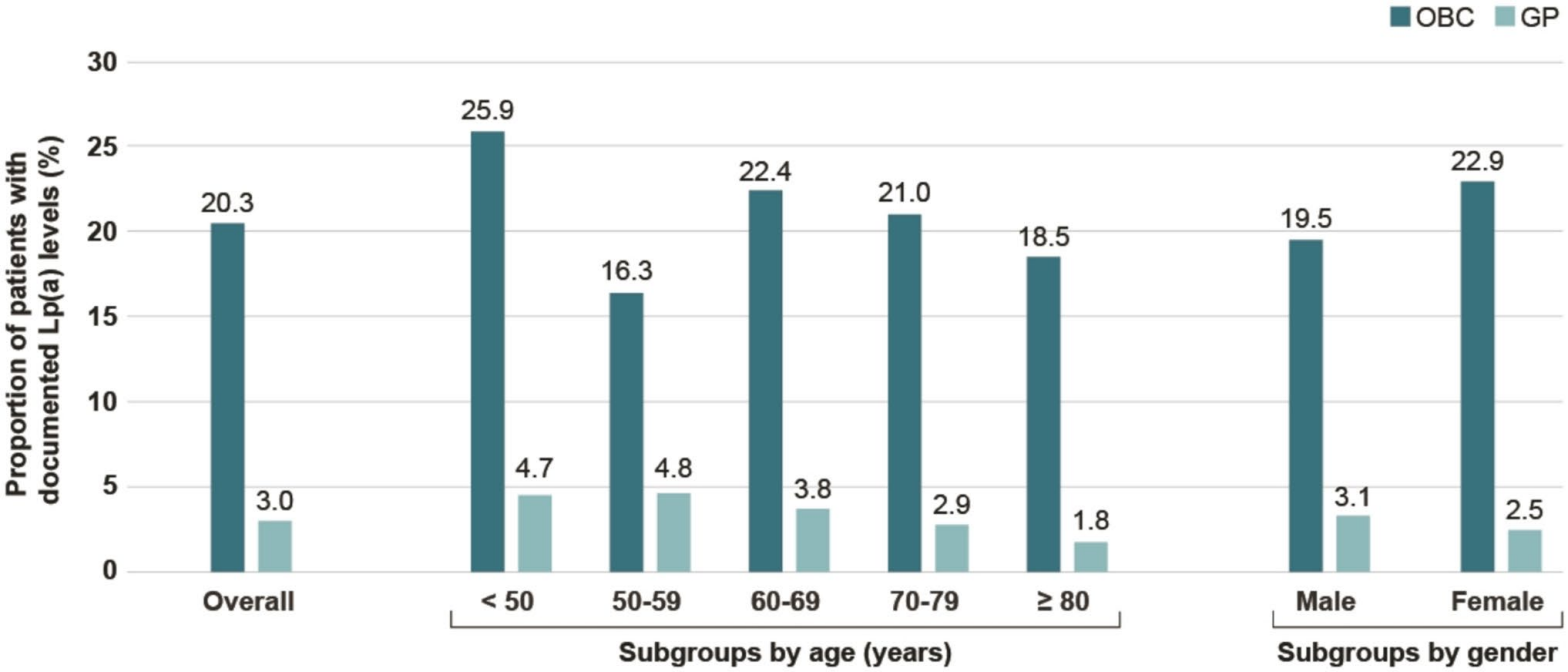
Proportion of patients with documented Lp(a) testing in the overall OBC and GP cohorts as well as by age subgroups and by gender subgroups

Lp(a) levels were documented in 20.3% of patients at OBCs compared to 3.0% at GPs. Testing rates tended to be higher in younger patients compared to older patients and were comparable in male and female patients in both the OBC and the GP settings (Fig. 3).

**Fig. 4:**
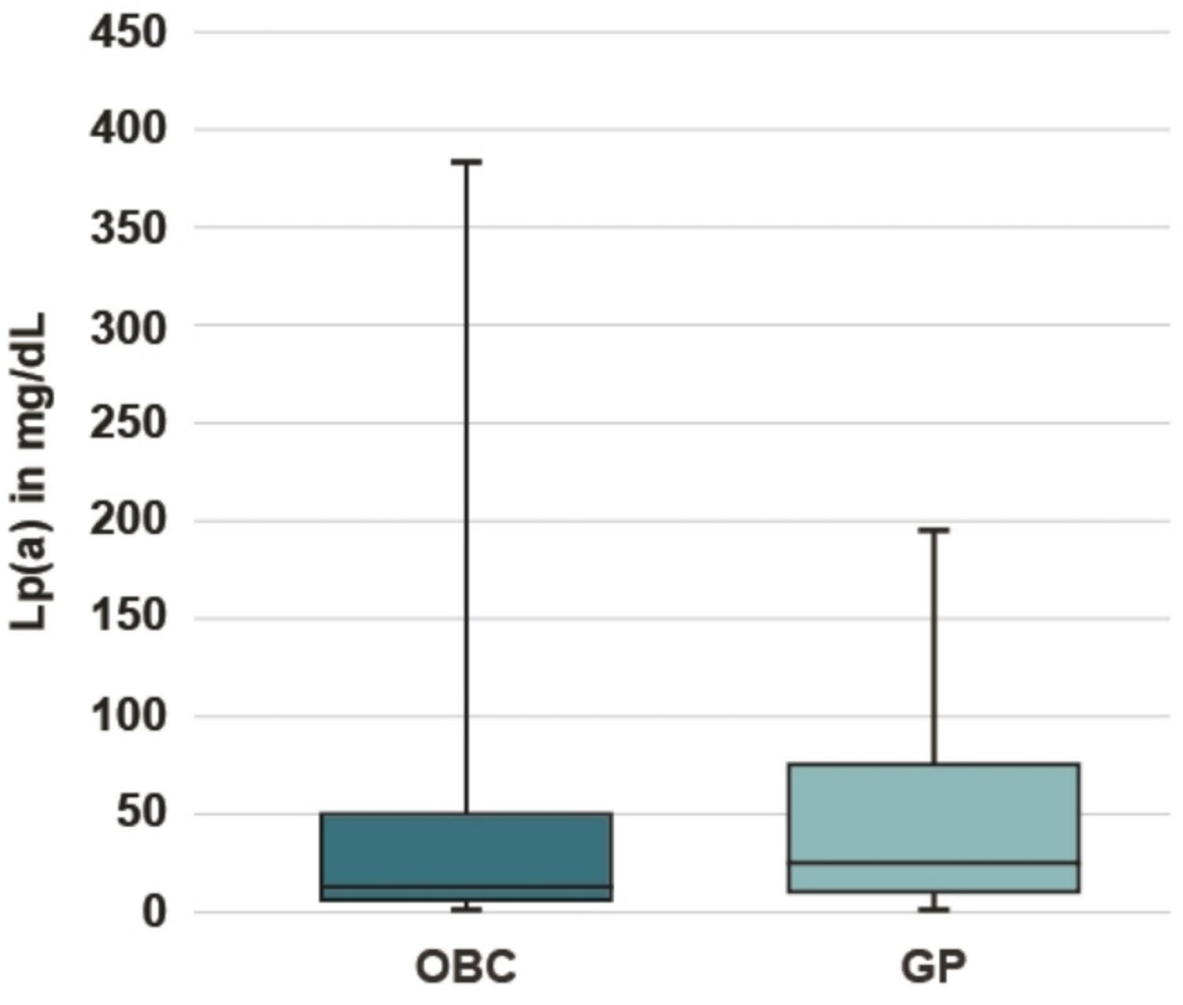
Box plot of Lp(a) levels in mg/dL (boxes indicate Q1 and Q3, whiskers indicate minimum and maximum values) in the OBC and GP cohorts

**Fig. 5:**
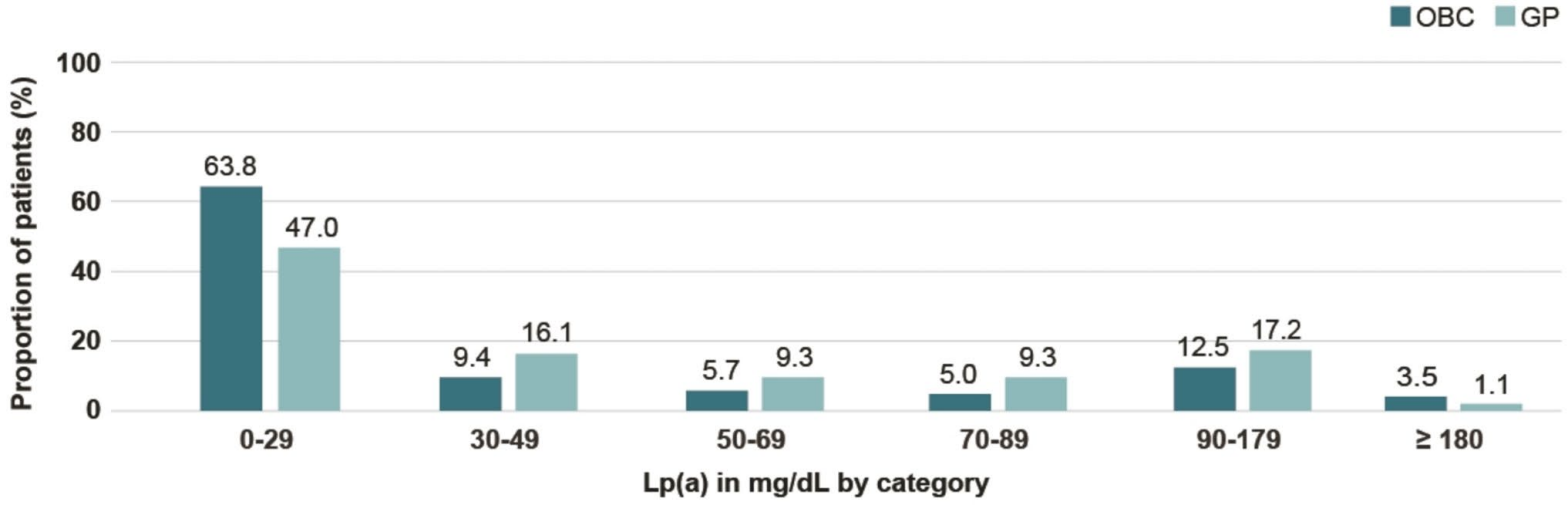
Proportion of patients by Lp(a) level (mg/dL) category in the OBC and GP cohorts

The median Lp(a) levels were 11 mg/dL in the OBC cohort compared to 24 mg/dL in the GP cohort (Fig. 4). Within the OBC cohort, 26.8% of patients had elevated Lp(a) levels ≥ 50 mg/dL compared to 36.9% in the GP cohort. Within the OBC setting, the proportion of patients with Lp(a) levels < 30 mg/dL was numerically higher (63.8%) compared to the GP setting (47.0%) (Fig. 5).

### Lipid-lowering therapy

**Fig. 6:**
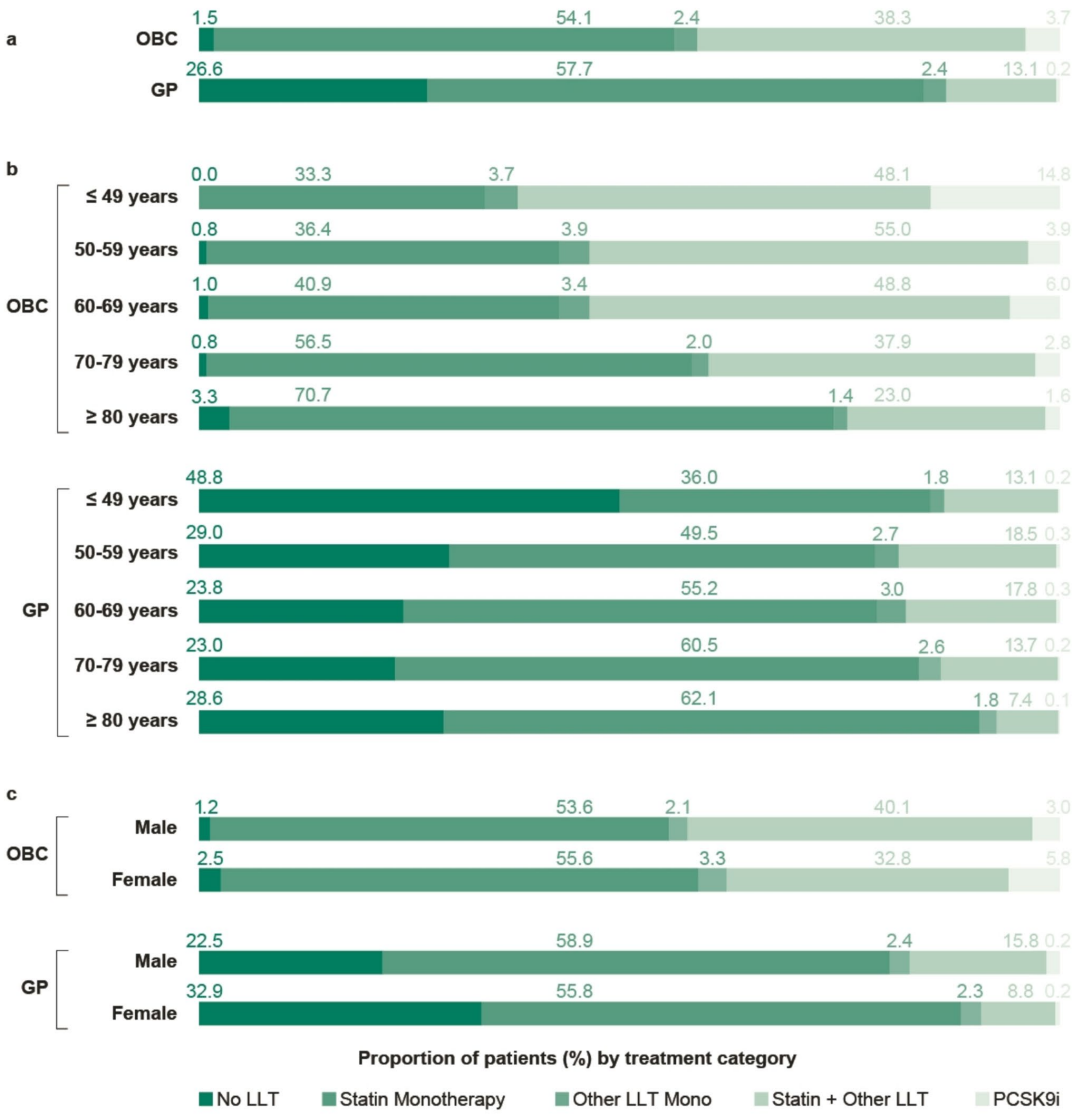
Proportion of patients by LLT choice in the total OBC and GP cohorts [a], in the OBC and GP cohorts by age subgroups [b], and in the OBC and GP cohorts by gender subgroups [c]. The category “Other LLT” includes ezetimibe, bempedoic acid, and/or bile acid sequestrants. The category “PCSK9i” includes monoclonal antibodies and inclisiran (monotherapy or combination with statins and/or other LLT)

Among patients in the OBC cohort, 1.5% received no LLT compared to 26.6% in the GP cohort (Fig. 6a), while 38.3% compared to 13.1% received statins plus other LLT (including ezetimibe, bempedoic acid, and/or bile acid sequestrants) (Fig. 6a).

None of the patients younger than 50 years in the OBC setting were untreated, whereas in the GP setting, 48.8% of patients in this age group received no LLT. In this age group, 33.3% (OBC) and 36.0% (GP) received statin monotherapy. The proportions of patients receiving statin monotherapy tended to be higher in the older compared to the younger age groups (Fig. 6b).

The proportions of male and female patients receiving no LLT were 1.2% vs. 2.5% in the OBC cohort and 22.5% vs. 32.9% in the GP cohort. For treatment with statin plus other LLT, the proportions were 40.1% vs. 32.8% (OBC) and 15.9% vs. 8.8% (GP) (Fig. 6c).

### Target achievement and Lp(a) testing rate by choice of lipid-lowering therapy

**Fig. 7:**
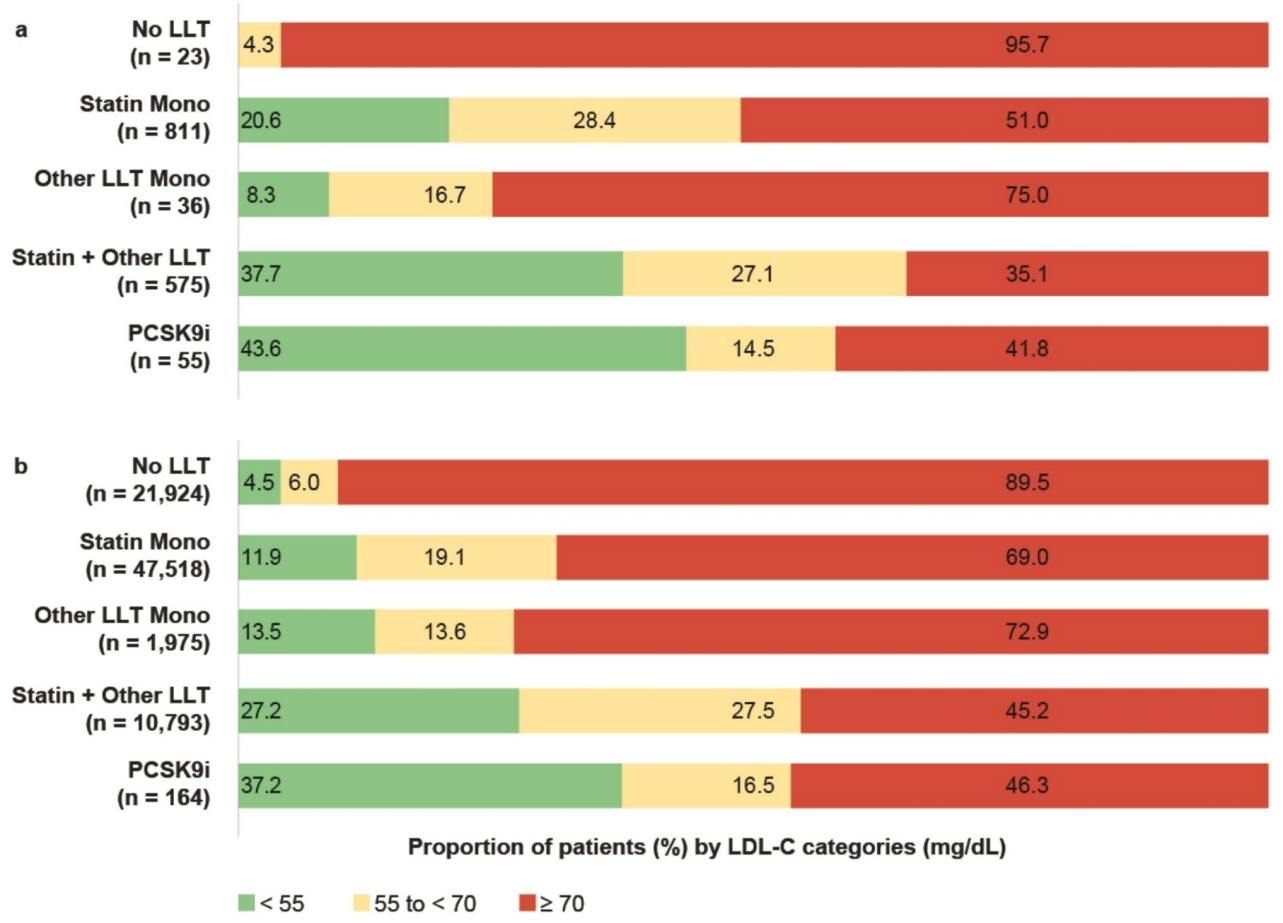
Proportion of patients by LDL-C category and LLT choice within the OBC [a] and GP [b] cohorts. The category “Other LLT” includes ezetimibe, bempedoic acid, and/or bile acid sequestrants. The category “PCSK9i” includes monoclonal antibodies and inclisiran (monotherapy or combination with statins and/or other LLT)

In the OBC cohort, none of the patients had LDL-C levels < 55 mg/dL in the absence of LLT compared to 4.5% in the GP cohort (Fig. 7a and b). Among patients undergoing treatment in the OBC cohort, the target achievement rate was 20.6% with statin monotherapy, compared to 43.6% with PCSK9i monotherapy or combination therapy (Fig. 7a). In the GP cohort, the target achievement rate (< 55 mg/dL) was 11.9% with statin monotherapy, compared to 37.2% with PCSK9i monotherapy or combination therapy (Fig. 7b). Considering a target level of < 70 mg/dL, 31.0% had achieved the target with statin monotherapy, compared to 53.7% with PCSK9i monotherapy or combination therapy in the GP cohort (Fig. 7b).

The highest Lp(a) testing rate was observed in patients receiving PCSK9i mono- or combination therapy in both settings, and in addition in those receiving other (non-statin) LLT monotherapy among OBC-documented patients (Table 3).

**Table 3:** Proportions of patients with documented Lp(a) level by LLT choice within the OBC and GP cohorts

	OBC N = 1,500		GP N = 82,375	
Treatment	n per subgroup	Lp(a) documented, %	n per subgroup	Lp(a) documented, %
No LLT	23	17.4	21,924	1.0
Statin monotherapy	811	18.7	47,518	3.1
Other LLT monotherapy	36	30.6	1,975	7.0
Statin + other LLT	575	20.3	10,793	5.8
PCSK9i	55	38.2	164	29.3

The category “Other LLT” includes ezetimibe, bempedoic acid, and/or bile acid sequestrants. The category “PCSK9i” includes monoclonal antibodies and inclisiran (monotherapy or combination with statins and/or other LLT)

## Discussion

The findings of LipidSnapshot indicate that the majority of ASCVD patients were not treated adequately, regardless of the treatment setting, age, or gender. However, some relevant health care differences were noted.

### Impact of the healthcare setting

Primarily, the discrepancies in LLT and LDL-C levels among patients documented by both OBCs and GPs highlight a significant structural gap in the treatment landscape of ASCVD patients in Germany. Patients documented by GPs exhibited higher LDL-C levels, including a larger proportion of patients with LDL-C levels ≥ 190 mg/dL, which are indicative of a familial hypercholesterolemia (FH). Due to the immense cardiovascular risk associated with FH, these patients should undergo genetic testing and should be appropriately managed to achieve significant LDL-C reduction [6]. Furthermore, a lower proportion of patients in the GP cohort reached the LDL-C target levels of < 55 mg/dL, as compared to those documented by OBCs. A similar discrepancy is observed regarding the achievement rates for an LDL-C target of < 70 mg/dL. Therapy intensification, including combination therapy, is more prevalent in the OBC setting.

Regarding the discrepancies in target achievement rates between OBCs and GPs, it is essential to consider that disparate targets are applicable to these settings. While the ESC/EAS guidelines [6]. which are relevant for OBCs, recommend LDL-C reduction of ≥ 50% from baseline and a LDL-C target level of < 55 mg/dL in ASCVD patients, the NVL [5]. which is one of the main guidelines for GPs, mentions both the “fire-and-forget” strategy as well as the “treat-to-target” strategy with a target LDL-C level of 70 mg/dL. The attainment rates for the targets specified within the context of each setting are comparable between OBCs (< 55 mg/dL: 27.4%) and GPs (< 70 mg/dL: 28.7%), suggesting the discrepancies in LDL-C target attainment are mainly due to the divergent recommendations in the guidelines.

In addition to these discrepancies in guideline recommendations, current reimbursement restrictions for PCSK9 modifying therapies may contribute to discrepancies between OBCs and GPs. The current findings indicate that only a small number of patients are treated with PCSK9 modifying therapies, almost none of which were observed in the GP setting. In Germany, these therapies must be initiated by specialists, including those in the fields of cardiology, nephrology, endocrinology, diabetology, and angiology, as well as by specialists in lipid outpatient clinics. Subsequently, a continuous prescription is also permissible in the GP setting. The low prescription rates observed in the GP setting suggest that patients who consult an OBC tend to remain in specialist care.

It is unfortunate that treatment options, including the prescription of PCSK9i, are not being utilized to their fullest potential, given the effectiveness of these options. A simulation study indicated that approximately half of patients in secondary prevention require PCSK9i to achieve target LDL-C levels [7]. In the “Jena auf Ziel” study, high-risk ASCVD patients received a high-intensity statin in combination with ezetimibe. Among these patients, 80% achieved the LDL-C target levels recommended by the ESC/EAS. Only 20% of the patients required escalation therapy with bempedoic acid or PCSK9i. In all patients receiving triple therapy the LDL-C target was achieved [8].

Overall, the discrepancies observed between the OBC and the GP setting prompt the question of whether comparable LDL-C targets and treatment strategies across the various guidelines would be beneficial, given that the same patients are involved irrespective of the structural settings. Enhanced cross-sectional networking could also potentially improve patient care.

### Age-specific disparities

Secondly, the present findings indicate age-specific disparities. Most strikingly, approximately 50% of patients younger than 50 years who were treated in a GP setting had not received any treatment despite their elevated cardiovascular risk. The majority of patients in both the OBC and GP settings have not achieved the target level of < 55 mg/dL. These findings are analogous to those observed in a cross-sectional study conducted at a Danish hospital among patients who experienced a cardiovascular event before the age of 40 years. In the hospital setting, approximately 60% of patients had not attained the ESC/EAS-recommended target level, which was defined as < 70 mg/dL at the time of the study [11]. It is notable that this cohort of young patients who experienced premature cardiac events, is at an elevated long-term risk. Although the relative one-year mortality risk does not appear to be age-dependent [13]. the longer remaining lifespan significantly elevates the absolute lifetime risk. Hereditary conditions such as FH or elevated Lp(a) levels frequently contribute to premature cardiovascular risk and complicate cardiovascular prevention [6]. Furthermore, it has to be kept in mind that the majority of patients with early ASCVD are engaged in paid employment. Cardiovascular re-events result in either temporary or permanent inability to work, which can also have economic consequences and result in costs to the healthcare and social system [14].

In addition, our data indicates that older patients were more likely to receive a statin monotherapy in both settings compared to younger patients. On the other hand, the proportion of patients who remained untreated increases once more in the group of patients aged ≥ 80 years. These findings align with those of a Swedish and a Canadian cross-sectional study, which both reported lower rates of LLT among patients aged ≥ 85 years compared to patients aged 75 to 84 years [15, 16]. The lower treatment rate in elderly patients may be attributed to concerns about adverse effects and drug interactions in a multimorbid population [17]. Additionally, the risk of a subsequent cardiovascular event within the remaining life expectancy may be considered less significant, leading to a less favourable benefit-risk balance [18].

### Gender gap

Thirdly, gender appears to influence lipid management in patients with ASCVD. In both treatment settings, LDL-C levels in men were observed to be numerically lower than in women, LDL-C levels indicative for FH were less common in men, and men were more likely to receive lipid-lowering treatment than women, indicating the presence of structural gender-related treatment differences.

These results are consistent with those of the EUROASPIRE IV cross-sectional study, initiated by the ESC. It was found that women were significantly less likely to achieve the LDL-C target than men and less likely to receive LLT. This was observed to be consistent across countries [12]. The EUROASPIRE study additionally indicates that the largest gender disparity was observed in patients with lower levels of education and advanced age. With advancing age and greater educational attainment, the gender gap diminished [19].

Previous research has indicated that lower adherence may be a potential explanation for the higher target failure rate observed in women [20]. Accordingly, a meta-analysis encompassing over 1.8 million elderly patients receiving statin treatment revealed that female gender was associated with nonadherence. The authors postulated that this may be attributable to heightened concerns about adverse effects among women or lower motivation to adhere to treatment due to underestimation of their cardiovascular risk [20]. As women overall tend to show greater concern for their health and engage more frequently with the health system than men [21]. the implementation of appropriate patient education may prove an effective means of reducing the gender gap.

### Rare testing of Lp(a)

Lp(a) was infrequently assessed, but more frequently in the OBC setting than in the GP setting and more frequently in patients receiving PCSK9i or non-statin LLT monotherapy. No discernible differences were identified based on age or gender, except for a slight tendency towards increased testing in younger patients. The ESC/EAS guidelines recommend Lp(a) testing, particularly in cases of premature ASCVD (Class IIa recommendation) [6]. In contrast, the NVL does not endorse Lp(a) testing [5]. which may explain the lower rates in the GP setting. However, although the ESC/EAS guidelines recommend measuring Lp(a) levels for each individual patient, this is not done as a standard procedure, even in younger patients in the OBC setting.

The present results are consistent with an analysis of claims data showing an Lp(a) testing rate of less than 2% even in younger patients with ASCVD [22]. A recent registry of cardiovascular rehabilitation clinics showed that Lp(a) is tested infrequently even in the context of major cardiac events, and at the same time the study demonstrated the above average prevalence of elevated Lp(a) levels in these patients [23]. The EAS/ESC Consensus Group has defined 50 mg/dL as relevant threshold for elevated Lp(a) levels [4]. According to the Copenhagen Heart Study, the prevalence of elevated Lp(a) levels above 50 mg/dL in the general European population is 20% [24]. In a cohort of 52,898 patients (not limited to patients with major cardiac events) admitted to a clinic for cardiology in Germany, 18.4% were reported to have Lp(a) levels above 50 mg/dL [25]. In our cohorts, the prevalence of elevated Lp(a) levels above 50 mg/dL is 26.8% in the OBC setting and 36.9% in the GP setting, higher in both settings than in the general population and in a cardiologic patient population not limited to those with major cardiac events.

In addition, the data revealed that the proportion of patients with levels below 30 mg/dL tended to be higher in the OBC cohort than in the GP cohort. The Lp(a) testing pattern suggests that OBCs and GPs may initiate Lp(a) testing in patients with different profiles. A lower overall prevalence of patients with Lp(a) levels below 30 mg/dL may be the result of a targeted approach to Lp(a) testing in the GP cohort. This focus is understandable as far as Lp(a) may be considered as a causal driver of ASCVD in patients with less remarkable elevation of LDL-C levels, patients with particularly early onset of ASCVD, or patients with recurrent events. Nevertheless, such a targeted approach may underestimate the Lp(a)-mediated risk in patients with a classic risk profile.

### Limitations

In order to accurately interpret the findings of the present study, it is essential to acknowledge the inherent limitations of the research design. It should be noted that the data were derived from disparate study parts, a prospective non-interventional study for OBC data and a retrospective analysis for GP data. The sample sizes between the two study parts differ considerably and the prospective OBC cohort is restricted to a sample size of 1,500 patients. This results in a relatively small number of subjects in some subgroups. Moreover, it should be noted that only 3.0% of the GPs and 20.3% of the OBCs within the panel provided Lp(a) levels. Missing data was not imputed. This lack of completeness in the Lp(a) data represents an additional limitation. In conclusion, the results can only be presented in a descriptive manner, and any interpretation should be regarded as hypothesis-generating.

## Conclusions

Overall, the results of LipidSnapshot highlight potential gaps in healthcare provision. A large proportion of ASCVD patients in Germany are inadequately treated, with notable differences between GPs and OBCs. Additionally, gender- and age-related disparities are evident. Given the efficacy of available LLT, the rates of target achievement in clinical practice are concerning. There is a clear need for these gaps to be addressed to improve cross-sectoral patient care.

## Data Availability

Data are available upon request from the corresponding author.
